# Standardization of the Teratoma Assay for Analysis of Pluripotency of Human ES Cells and Biosafety of Their Differentiated Progeny

**DOI:** 10.1371/journal.pone.0045532

**Published:** 2012-09-25

**Authors:** Michal Gropp, Vitali Shilo, Gilad Vainer, Miri Gov, Yaniv Gil, Hanita Khaner, Limor Matzrafi, Maria Idelson, Juri Kopolovic, Naomi B. Zak, Benjamin E. Reubinoff

**Affiliations:** 1 The Hadassah Human Embryonic Stem Cell Research Center, The Goldyne Savad Institute of Gene Therapy and Department of Gynecology, Hadassah-Hebrew University Medical Center, Jerusalem, Israel; 2 CellCure NeuroSciences Ltd., Jerusalem, Israel; 3 The Department of Pathology, Hadassah-Hebrew University Medical Center, Jerusalem, Israel; University of Sao Paulo - USP, Brazil

## Abstract

Teratoma tumor formation is an essential criterion in determining the pluripotency of human pluripotent stem cells. However, currently there is no consistent protocol for assessment of teratoma forming ability. Here we present detailed characterization of a teratoma assay that is based on subcutaneous co-transplantation of defined numbers of undifferentiated human embryonic stem cells (hESCs) with mitotically inactivated feeder cells and Matrigel into immunodeficient mice. The assay was highly reproducible and 100% efficient when 100,000 hESCs were transplanted. It was sensitive, promoting teratoma formation after transplantation of 100 hESCs, though larger numbers of animals and longer follow-up were required. The assay could detect residual teratoma forming cells within differentiated hESC populations however its sensitivity was decreased in the presence of differentiated cells. Our data lay the foundation, for standardization of a teratoma assay for pluripotency analysis. The assay can also be used for bio-safety analysis of pluripotent stem cell-derived differentiated progeny.

## Introduction

Human embryonic stem cells (hESCs) are pluripotent cells derived from preimplantation embryos. These cells can self-renew for long periods, and have the potential to differentiate into any cell type [1], [2]. Hence they may be exploited for applications in regenerative medicine, drug discovery and basic research of early human development.

When transplanted into immune-deficient mice at growth-permissive sites, hESCs form teratoma-like masses containing differentiated progeny representing the three germ layers [1]–[4]. Formation of cells from all three germ layers within a teratoma is considered as an essential criterion to define the pluripotent potential of a hESC line [5]. In the consensus guidance for the banking, testing and distribution of hESC lines, published by the International Stem Cell Banking Initiative (ISCBI), the teratoma formation assay is defined as the “gold standard” for pluripotency [6]. In accordance with these guidelines many publications on the derivation of new hESC lines include characterization of the line’s pluripotency by a teratoma formation assay. In addition, the teratoma assay is considered by many as a criterion that should be fulfilled in order to ascertain that a genuine induced pluripotent stem (iPS) cell line has been obtained [7]. However, the methods that different research groups use to induce teratomas differ in important parameters such as cell preparation, number of transplanted cells, mode of transplantation, site of transplantation, and the length of time that animals are monitored for tumor formation. Moreover, the pathological analysis of tumors and the report of experimental data are often incomplete and highly variable. If the teratoma assay is regarded as the “gold standard” for defining pluripotency, standardization of the assay is invaluable. Indeed, recently Mueller and colleagues published a call for the standardization of the teratoma formation assay [8].

Standardization of the teratoma assay is not important only for assessing hESC pluripotency but also for evaluating the tumorigenic potential of hESC-derived progeny. The field of hESCs is moving rapidly towards clinical applications, with the first spinal injury patient being recently transplanted with hESC derived cells [9]. A key hazard in the implementation of hESC-based cell therapy is potential tumor formation caused by the presence of pluripotent hESCs within the transplanted cell preparations. A standardized sensitive teratoma assay to detect low numbers of tumor forming cells within a therapeutic cell preparation would be highly valuable.

Previous studies reported inconsistent detection sensitivities of various teratoma assays. The sensitivities ranged from 1×10^4^ hESCs after intra-testicular [10], or intra-muscular [11] transplantation, 1×10^3^ after injection into human fetal tissue grafts in SCID mice [12], and 245 hESCs after intra-muscular co-injection of hESCs with their feeder fibroblasts [13].

In view of the importance of standardizing the teratoma assay, we present here a detailed characterization of an efficient, quantitative, sensitive and easy-to-perform teratoma assay. Our data lay the foundation, for the standardized use of this assay for the analysis of pluripotency of hESC and iPS cell lines. We further demonstrate the use of the assay for safety analysis of the tumorigenic potential of hESC-derived differentiated populations intended for the clinic.

## Results

### Key Features of the Teratoma Assay

To establish a teratoma assay that is sensitive, quantitative, easy to perform and to monitor, we included the following components in our protocol:

#### (1) Quantification of the number of transplanted cells

Prior to inoculation, hESC colonies were dissociated into single cell suspensions, to enable transplantation of defined numbers of cells.

#### (2) Characterizing the phenotype and genotype of transplanted cells

Prior to transplantation, we quantified by FACS the percentage of cells expressing pluripotency-associated cell surface markers (Tra-1-60, Tra-1-81, and SSEA-4). We also confirmed that the karyotype of the transplanted cells was normal.

#### (3) The mode and site of transplantation

To increase the sensitivity of the assay, hESCs were co-transplanted with mitotically inactivated human foreskin- fibroblast-feeders. The beneficial effect of co-transplantation of hESCs with their own feeders was observed by Hentze *et al* [13]. To further increase the sensitivity, the transplanted cells were also mixed with Matrigel. The cells were injected subcutaneously (s.c.) since transplantation to this site is easy to perform, does not involve an invasive surgical procedure, and allows simple monitoring of teratoma development. Subcutaneous transplantation of hESCs with Matrigel was reported to induce teratomas with high efficiency [14].

#### (4) Recipient animals

To minimize the risk of immune rejection, the cells were transplanted into NOD/SCID mice. These mice were previously shown to tolerate hESC grafts well in comparison to immune-competent mice [15]. Since NOD/SCID mice have a tendency to develop spontaneous tumors, and to rule out the unlikely formation of tumors by the co-transplanted feeders, in each experiment we included a control group of mice transplanted with mitotically-inactivated feeders.

#### (5) Duration of monitoring of tumor formation

We monitored the development of tumors in the transplanted animals for a period of 30 weeks. The prolonged follow-up increased the sensitivity of the assay by allowing the detection of late appearing tumors that developed from a small number of cells.

#### (6) Defined histological criteria for teratoma

We defined a tumor as a teratoma only if it contained tissues representing all three germ layers. The histological analysis was performed by a pathologist.

We adhered to these guidelines throughout the study. We characterized the sensitivity of detection and the time course of tumor formation with this teratoma assay protocol by transplanting various numbers of hESCs, ranging from 5×10^5^ to 1×10^1^. We also studied whether the efficiency and sensitivity of the assay could be improved by supplementing the transplanted cell preparation with the p-160-Rho-associated coiled-coil kinase (ROCK) inhibitor, Y-27632. This inhibitor was shown to enhance hESC survival under unfavorable conditions [16]. We thus hypothesized that its addition to the dissociated hESCs prior to transplantation might have a beneficial effect on their survival and improve the efficiency of teratoma formation from a small number of transplanted hESCs.

### Evaluation of the Assay with a Relatively Large Number of Transplanted Cells

**Figure 1:**
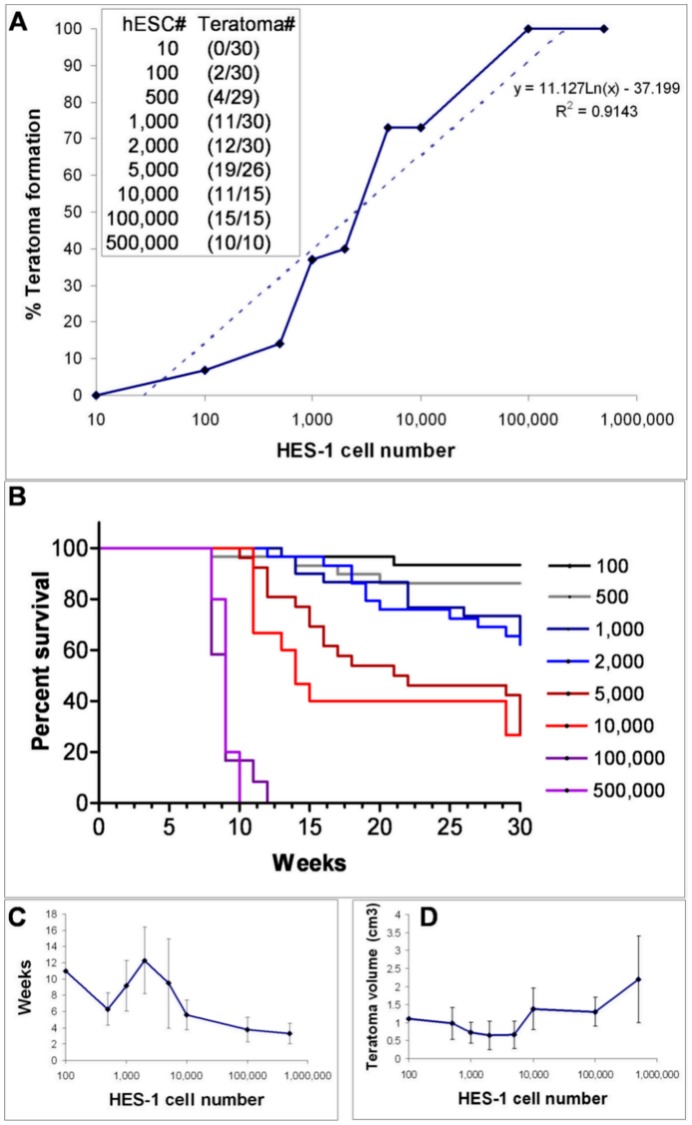
Kinetics of Teratoma Formation after Transplantation of Various Specific Numbers of Undifferentiated HES-1 Cells. Specific numbers of undifferentiated HES-1 cells were mixed with MMC-treated foreskin fibroblasts (to a total of 1×10^6^ cells) and Matrigel, and transplanted s.c. into NOD/SCID mice. The transplanted animals were monitored weekly for the appearance of tumors, and for the progression of tumor size. The endpoint of the experiments was when the tumors reached a size of ≥ 1 cm^3^ or 30 weeks after transplantation. (**A**): Efficiencies of teratoma tumor formation after transplantation of decreasing numbers of undifferentiated HES-1 cells. The trendline is depicted in dotted line. (**B**): A Kaplan-Meier plot showing the percentage of surviving mice transplanted with decreasing numbers of hESCs, at various time-points (1 W–30 W) during the transplantation experiments. The mice were sacrificed when the tumors reached a volume of ≥ 1 cm^3^. (**C**): The average time interval between transplantation and the detection of tumors. (**D**): The average volume of the teratomas at the time of animal sacrifice. All data relate to animals that developed teratomas. Data presented as mean ± SEM.

**Figure 2:**
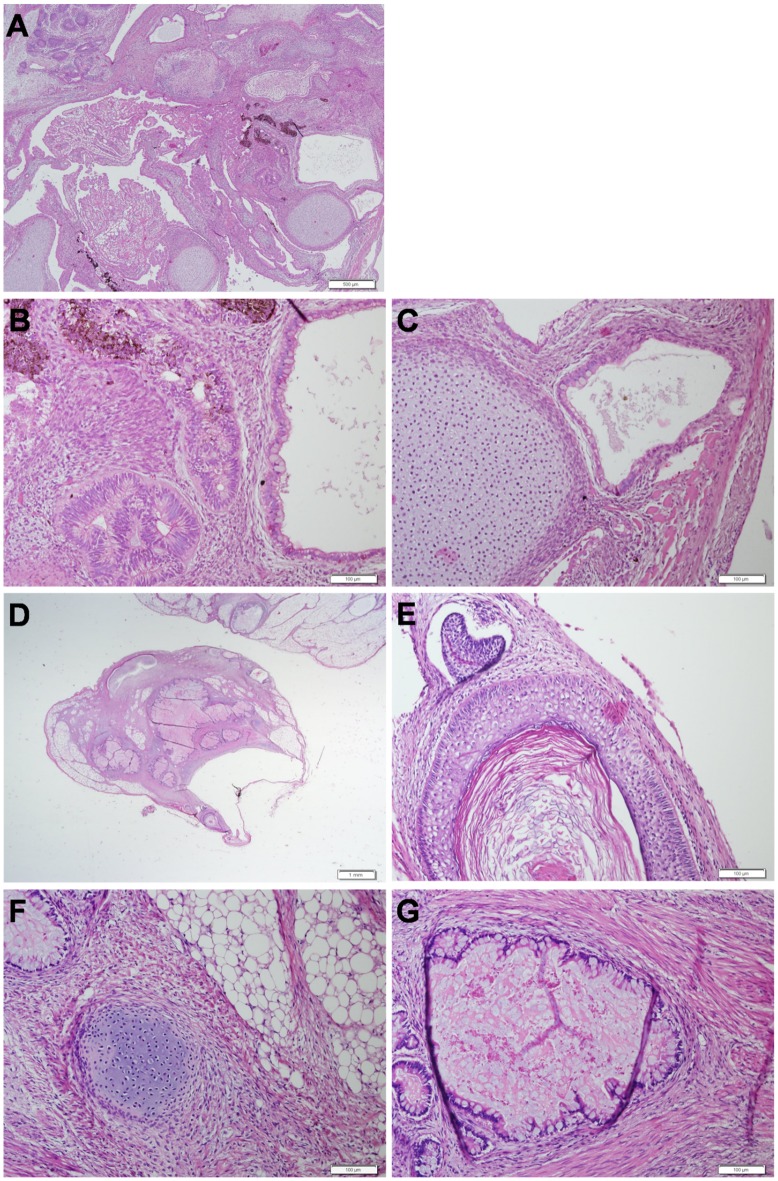
Histological Analysis of Teratomas Formed after Transplantation of undifferentiated HES-1 Cells. Images of sections of teratomas, formed after s.c. transplantation of undifferentiated hESCs with their feeders and Matrigel into NOD/SCID mice. (**A–C**): A teratoma formed from transplantation of 5×10^5^ hESCs. (A) A lower magnification (× 4) of a section of a teratoma showing derivatives of all three germ layers. (B–C) A higher magnification (× 20) of regions of (A) showing ectodermal, and endodermal structures (B) and mesodermal and endodermal structures (C). (**D–G**): A teratoma formed from transplantation of 100 hESCs. (D) A lower magnification (× 1.25) of a section of a teratoma showing derivatives of all three germ layers (E–G) a higher magnification (× 20) of regions of (D) showing ectodermal structures (E), mesodermal structures (F) and endodermal structures (G).

In initial experiments we evaluated the assay by transplantation of relatively large numbers of undifferentiated hESCs (5×10^5^ and 1×10^5^). Prior to transplantation we confirmed that the transplanted hESCs (HES-1 cell line; [2]) carried a normal karyotype (46, XX). FACS analyses, performed on the day of transplantation, showed that the majority of transplanted cells expressed high levels of cell surface markers associated with undifferentiated hESCs (Tra-1-60, Tra-1-81, and SSEA-4: Fig. S1A). Five week old NOD/SCID mice (n = 10) were transplanted subcutaneously with 5×10^5^ hESCs combined with 5×10^5^ mitomycin (MMC)-treated foreskin cells, and Matrigel, in the presence or absence of the ROCK inhibitor Y-27632 (5 animals in each group). In all transplanted animals, tumors were observed 3–4 weeks after transplantation. By 8–10 weeks the tumors were larger than 1 cm^3^ (average tumor volume of 2.2±0.4 cm^3^) and the animals were sacrificed (Fig. 1). Macroscopic analysis of the tumors revealed that they were encapsulated, and sometimes cystic. Assessment of the peritoneal cavity, liver, spleen and lungs tissues of the sacrificed animals did not show additional sites of tumor formation. Histological analysis of each of the tumors revealed the presence of cells and structures representing the three germ layers. The three germ layer structures were randomly arranged within the teratomas (Fig. 2 A–C). In contrast, none of the control animals (n = 10) transplanted with 1×10^6^ MMC-treated foreskin fibroblasts, either in the presence or absence of ROCK inhibitor, developed tumors. Notably, the addition of ROCK inhibitor had no effect on the length of time from injection to tumor detection, nor did it promote the development of larger teratomas (Fig. S2).

Transplantation of 1×10^5^ hESCs gave similar results. All animals (n = 15) transplanted with hESCs developed teratomas while none of the control animals (n = 6) developed tumors. The tumors were first observed 3–5 weeks after transplantation and reached volumes larger than 1 cm^3^ by 8–9 weeks (average tumor volume of 1.3±0.1 cm^3^) (Fig. 1). Addition of ROCK inhibitor had no effect on the timing of tumor detection or on its size (Fig. S2).

Transplantation of 1×10^5^ hESCs from a different line (HES-2, [2]) also gave rise to teratomas in all animals (n = 3) (Table S1).

These results demonstrated that subcutaneous transplantation of 5×10^5^ or 1×10^5^ undifferentiated hESCs, combined with their feeders and Matrigel, into NOD/SCID mice was highly efficient, leading to teratoma formation in 100% of the transplanted mice. Moreover, tumor progression was rapid, as shown by the detection of tumors in all animals within 3.7±0.3 weeks, and the development of teratomas to a size larger than 1 cm^3^ within 9.0±0.2 weeks after transplantation.

### Determination of Assay Sensitivity

After demonstrating that our assay could efficiently promote teratoma formation from relatively large numbers of hESCs, we sought to evaluate the sensitivity of the assay. For this purpose we transplanted decreasing numbers of undifferentiated HES-1 cells (1×10^4^, 5×10^3^, 2×10^3^, 1×10^3^, 5×10^2^, 1×10^2^, and 1×10^1^) using the transplantation method described above. Since we expected lower efficiencies of teratoma formation after transplantation of decreased numbers of hESCs, we increased the size of the transplanted animal groups (15–30 animals/group) per each specific number of hESCs. In addition, in each transplantation experiment, we included f a positive control of a mouse transplanted with 1×10^5^ hESCs. In accordance with our protocol, we first confirmed that the majority of transplanted hESCs expressed high levels of pluripotency associated cell surface markers (Fig. S1B) and had a normal karyotype. All the positive control animals (n = 6), transplanted with 1×10^5^ hESCs, rapidly developed teratomas, while we did not observe tumors in any of the negative control animals (n = 30), transplanted with 1×10^6^ feeders cells (in the presence; n = 15 and absence; n = 15 of ROCK inhibitor). As the number of transplanted hESCs decreased we observed lower efficiencies of teratoma formation (Fig. 1A). The smallest number of undifferentiated HES-1 cells that could still generate teratomas in our assay was 100 hESCs (Fig. 2 D–G). We did not observe tumor formation in animals transplanted with 10 hESCs. When we subjected the data in Fig. 1A to a regression analysis, a trendline with a high R squared value (R^2^  =  0.91) was obtained. This analysis revealed that the predicted detection limit of the assay is 28 hESCs (Fig. 1A). Decreased numbers of transplanted hESCs affected not only teratoma formation efficiency but also the kinetics of teratoma formation. When lower numbers of hESCs were transplanted, the time intervals for tumor detection were prolonged (Fig. 1C). A Kaplan-Meier analysis of the fraction of the animals that survived at different time points along the experiment (1 W–30 W) revealed a significant increase in the fraction of surviving animals (p< 0.0001) as the number of transplanted hESCs decreased (Fig. 1B). Moreover, when 5,000 or less hESCs were transplanted in many animals the tumors did not reach a volume of 1 cm^3^ by 30 weeks (Fig. 1D). In addition, histological analysis showed that with low numbers of transplanted hESC (≤5,000 cells) some small tumors (8 tumors with average volume of 0.3±0.12 cm^3^) did not include derivatives of all three germ layers, and therefore did not meet the histological criteria of a teratoma tumor. These non-teratoma tumors were excluded from the analysis.

We confirmed with an additional line (HES-2) that the efficiency of teratoma formation decreases with the reduction of the number of transplanted cells, and that transplantation of 100 cells could give rise to teratoma formation (Table S1).

These results demonstrate that the assay is highly sensitive, promoting and detecting teratoma formation by as few as 100 undifferentiated hESCs.

### Effect of ROCK Inhibitor on Teratoma Formation

As mentioned above, for each experimental group with a specific number of hESC, cells were transplanted in the presence or absence of ROCK inhibitor. Given the improved survival of dissociated hESCs in the presence of ROCK inhibitor *in vitro* [16], we tested whether its supplementation would improve the efficiency of teratoma formation, in particular when a small number of hESCs are transplanted. We did not observe any beneficial effect of ROCK inhibitor supplementation, neither on tumor formation efficiency nor on tumor growth (Fig. S2). Since the ROCK-inhibitor did not increase the sensitivity of the assay we did not include it in the following experiments.

### Using the Assay to Detect Teratoma Forming Cells within hESC-derived Differentiated Cell Populations

We next analyzed the capability of the assay to evaluate the tumorigenic potential of hESC-derived differentiated populations. We evaluated the assay with two types of differentiated cell populations that differed with regard to their contamination by undifferentiated hESCs.

A population of differentiated cells enriched for neural progenitors (NPs) was derived from hESCs (HES-1) as previously described [17]. Routine FACS analysis performed after 6 weeks of differentiation showed that the majority of cells (94.6 ± 1.5%) expressed the early neural marker PSA-NCAM, while a minority of the cells (3.2 ± 0.8%) expressed the pluripotent stem-cell marker Tra-1-81. Following transplantation of 5×10^5^ cells from 7 W NPs enriched population, mixed with 5×10^5^ feeders, 1 out of 4 mice (25%) developed a teratoma. These results confirmed the ability of the assay to detect residual pluripotent stem cells within differentiated progeny of hESCs.

**Figure 3:**
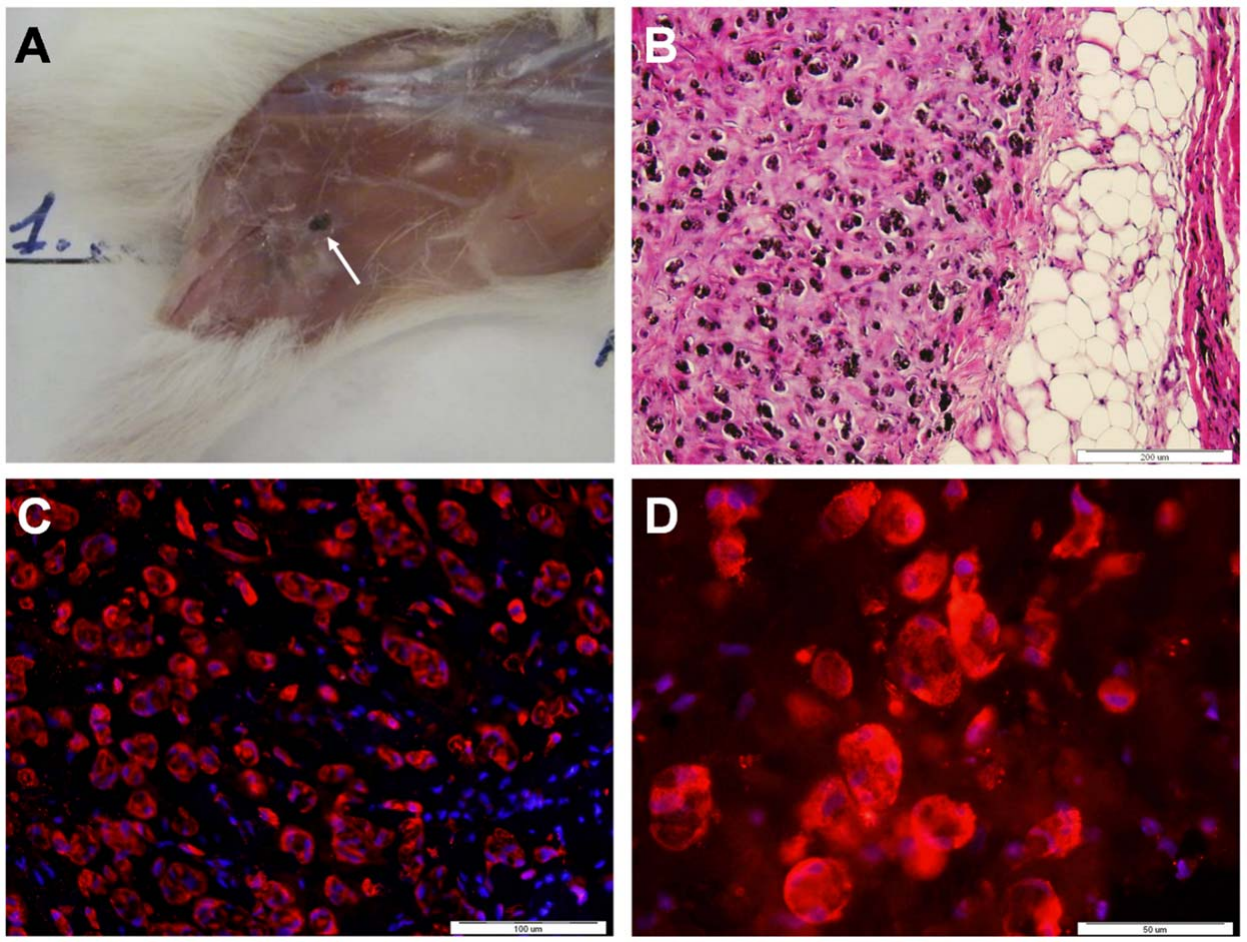
Analysis of HES-1-derived RPE Cell Grafts. Following transplantation of 5×10^5^ HES-derived differentiated RPE cells, the transplanted cells remained as small RPE-grafts at the site of injection. (**A**): The grafts could be visualized because RPE cells are pigmented. The arrow indicates the pigmented graft. (**B**): H&E staining of a section of a RPE-graft revealed pigmented cells. (**C–D**): Immunofluorescence staining of a section of the graft for markers specific for RPE cells, Bestrophin (C, red), and RPE65 (D, red), together with DAPI (blue) showed that the majority of cells within the graft expressed these markers.

We next transplanted RPE cells that were derived from HES-1 cells according to our previously published protocol [18]. FACS analyses, performed on the day of transplantation, showed that the majority of transplanted cells (99%) expressed high levels of CD81, a cell surface marker associated with RPE cells [19], while cells expressing Tra-1-60 and Tra-1-81 were not detected. 15 animals were transplanted s.c., in two separate experiments, with 5×10^5^ hESC-derived RPE cells, mixed with 5×10^5^ MMC-treated foreskin fibroblasts, and Matrigel. After an observation period of 30 weeks, we did not detect teratomas in any of the transplanted animals. The transplanted RPE cells remained at the site of transplantation as small grafts that could be visualized due to their pigmentation (Fig. 3A). Histological analysis of the RPE-grafts revealed that they contained pigmented cells (Fig. 3B). Imunofluorescence staining of a representative RPE-graft showed that the majority of the cells within the graft expressed high levels of Bestrophin (Fig. 3C) and RPE65 (Fig. 3D), markers specific for RPE cells. Thus the transplanted RPE cells survived at the site of transplantation, and in line with the FACS analysis that did not show residual pluripotent stem cells, teratoma tumors were not observed.

**Figure 4:**
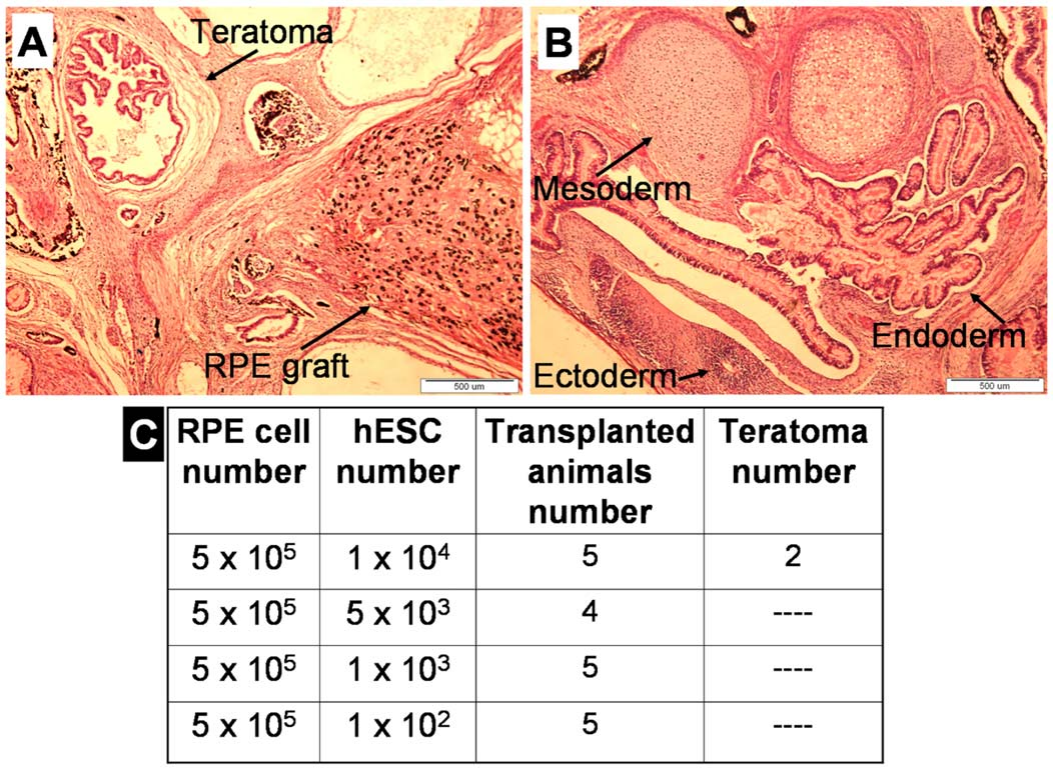
Histological Analysis of Teratomas formed after Transplantation of RPE Cells Spiked with Undifferentiated HES-1 Cells. (**A–B**): H&E staining of a section of a teratoma and an RPE graft generated after transplantation of 5×10^5^ HES-1-derived RPE cells spiked with 1×10^4^ undifferentiated HES-1 cells. (**C**): Efficiency of teratoma formation after transplantation of 5×10^5^ HES-1-derived RPE cells spiked with decreasing numbers of undifferentiated HES-1 cells.

We further performed a spiking study to analyze the sensitivity of the assay to detect pluripotent stem cells within the RPE population. We spiked decreasing numbers of undifferentiated HES-1 cells into 5×10^5^ HES1-derived RPE cells mixed with 5×10^5^ MMC-treated foreskin fibroblasts. Spiking of 1×10^4^ undifferentiated HES-1 cells into 5×10^5^ RPE cells promoted teratoma formation adjacent to a small RPE graft (Fig. 4A–B) in 2 out of 5 transplanted animals. Spiking of lower numbers of hESCs (5×10^3^, 1×10^3^, and 1×10^2^) did not generate teratomas (Fig. 4C), and the transplanted RPE cells generated only the small pigmented grafts.

Thus, the sensitivity of the assay was lower when pluripotent hESCs were transplanted in the presence of RPE cells.

## Discussion

The field of pluripotent stem cells is rapidly evolving concomitant with the establishment of multiple new hES and iPS cell lines worldwide. Defining uniform standards for the assessment of the pluripotency of new putative pluripotent stem cell lines is of major importance. The teratoma assay is considered by many as one of the minimal criteria that should be fulfilled in order to determine pluripotency [7], [8], [20]. However, currently there is lack of consistency in the performance and analysis of the assay in different laboratories.

Here we present a quantitative analysis of a teratoma assay in which defined numbers of characterized hESCs were co-transplanted subcutaneously with feeders and Matrigel into NOD/SCID mice. We show that the assay was highly reproducible and 100% efficient when 1×10^5^−5×10^5^ hESCs were transplanted, with decreasing efficiency when smaller numbers of hESCs were transplanted. We further show that the assay is sensitive down to 1×10^2^ hESCs. Importantly, we demonstrate the potential usefulness of this assay for bio-safety detection of residual teratoma forming cells within differentiated cell populations, albeit with a lower sensitivity than when pure hESCs are transplanted. Our data pave the way towards standardization of the teratoma assay for analysis of pluripotency and for analysis of residual teratoma forming ability in therapeutic cell populations during product development.

In the design of the assay we specifically addressed parameters which we considered important for standardization of the teratoma assay. These included characterization of the transplanted stem cells, quantification of the number of engrafted cells, the mode and site of transplantation, monitoring of tumor formation and growth, and its pathological analysis. The assay was designed on the basis of pooled published data with emphasis on the approach that it should be easy to perform and monitor and consistently promote efficient formation of teratomas.

The phenotype and genotype of the transplanted cells may affect the results of the teratoma assay. hESC cultures are most commonly heterogeneous with variable levels of background differentiation. Transplantation of relatively homogenous pluripotent stem cell populations is expected to promote higher efficiencies of teratoma formation. Therefore, since the goal of this study was standardization of the assay, we confirmed that the vast majority of transplanted cells in the various experimental groups expressed pluripotency-associated cell-surface markers. Quantitative analysis by FACS of the percentage of undifferentiated cells within transplanted cell preparations should be routinely included as part of the teratoma assay. Prior to transplantation we also confirmed the normal karyptype of the transplanted cells. During prolonged propagation in culture hESC and iPS cell lines were shown to acquire chromosomal abnormalities of which some are similar to those observed in their malignant counterparts, embryonal carcinoma (EC) cells [21]–[24]. Similar to EC cells, karyotypically abnormal hESCs and iPS cells may generate teratocarcinomas that contain undifferentiated cells [24], [25] and show more limited differentiation capacity. Hence, it is essential to ascertain that stem cells that are evaluated by the teratoma assay carry a normal diploid karyotype.

Immune deficient NOD/SCID mice were used in our assay since they tolerate hESC grafts and support the development of teratoma tumors [2], [15]. A potential disadvantage of NOD/SCID mice is their tendency to develop thymus tumors. However, within ten weeks of follow up, none of the transplanted mice developed these tumors. Moreover, none of the control mice that were grafted only with fibroblasts developed tumors. These data support the suitability of this animal model for the standardized teratoma assay of pluripotency.

A qualitative histological analysis of the tumors was performed by a certified pathologist. This ensured accurate identification of progeny representing the three germ layers.

As part of assay development, we determined the sensitivity of the assay that included all the above parameters. We carried out quantitative analysis of the ability to detect teratomas after transplantation of decreasing hESC numbers. We show that transplantation of 1×10^5^−5 hESCs reproducibly gave rise to teratoma tumors that developed into a size of 1 cm^3^ within 10 weeks in all transplanted animals. Given the 100% reproducibility of the assay with this cell number, if the number of cells to be transplanted is within or above this range, a limited number of animals may be used for the analysis of pluripotency.

**Figure 5:**
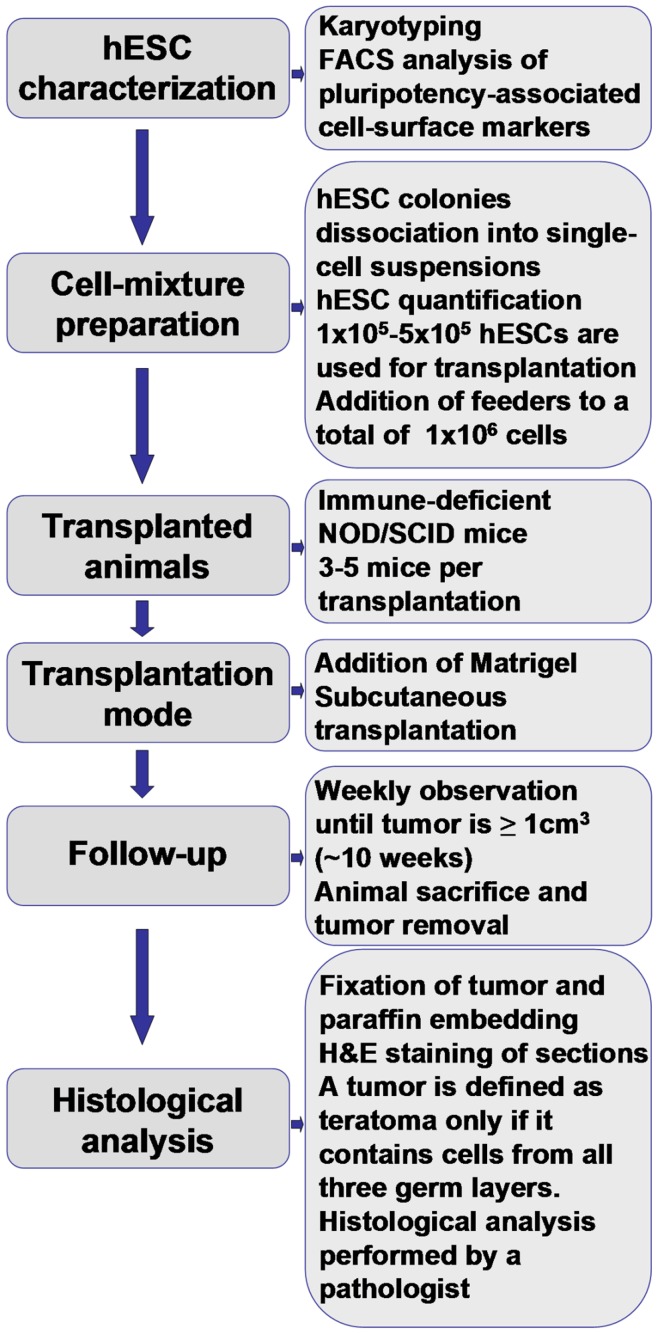
Proposed protocol for a standardized teratoma assay of pluripotency. The scheme describes our recommendations for the performance and analysis of a standardized teratoma assay of pluripotency.

Based on the data presented, Figure 5 shows our recommendations for a standard teratoma assay for pluripotency.

The standardized assay presented here is based on s.c. injection and is easy to perform and monitor. It is highly sensitive, allowing the detection of as few as 1×10^2^ undifferentiated hESCs. A quantitative limiting dilution analysis of the data in Fig. 1A suggested that the detection limit of the assay is 28 hESCs. This sensitivity is higher than the reported sensitivity in previous publications [11]–[13] and may be attributed to the site of transplantation as well as the inclusion of both feeders [13] and Matrigel [14] in the transplanted cell-mixture. In light of the low number of hESCs that generated teratomas in our xenograft transplantation assay, the possibility that even a lower number of residual pluripotent cells may give rise to teratomas in an autologous iPS transplantation should be considered. In the mouse ES cell system, the minimal number of ES cells that could generate teratomas was reported to be 2 cells [26]. The higher sensitivity of the mESC assay could be due to a higher tumorigenic potential of mESCs [24], or due to a higher survival rate of mESCs as single cells. The fact that the inclusion of ROCK inhibitor in the transplants did not increase the appearance of tumors, suggests that the former may be the more likely reason.

Assessment of the bio-safety of stem cell-based products for clinical studies and future therapy is of major importance, and several publications have proposed guidelines for the design of safety assays in animal models [27], [28]. It is largely accepted that safety analysis should include evaluation of the tumorigenic potential of the cells after transplantation into the intended site of clinical transplantation, using methods of delivery that mimic, as best as possible, the planned clinical study [27]. Nonetheless, a universal, sensitive, quantitative and easy to perform teratoma assay such as described here may be highly valuable for on-line assessment of the tumorigenic potential of hESC-derived differentiated cell preparations during the course of product development. Indeed, we found a good correlation between the ability or inability of differentiated populations of NP or RPE, respectively, to bring about teratoma formation under these assay conditions and other characteristics of each population pointing to the presence, or absence, of pluripotent cells.

Our data show that tumors arising from the transplantation of low numbers of undifferentiated hESC may be detected after a prolonged monitoring period and at relatively low frequencies. Therefore, long-term follow-up, and expanded animal experimental groups are needed to detect low numbers of undifferentiated tumor forming cells within differentiated cell preparations. Several lines of evidence indicate that the potential of undifferentiated hESCs to give rise to teratoma tumors was reduced when they were transplanted in the presence of a mixture of differentiated cells and feeders compared to feeders only. This was observed for the residual, about 3%, undifferentiated hESCs present within a cell population enriched for NPs. In this experiment the efficiency of tumor formation by about 15,000 hESCs among the 5×10^5^ NP cells (25%) was lower than anticipated (about 70%). It was further observed in the RPE spiking experiments in which the presence of 1×10^4^ undifferentiated hESCs added to 5×10^5^ RPE cells gave rise to teratoma formation in 40% of the cases but the presence of a smaller number of hESCs, such as 5×10^3^, did not yield any teratomas. The reduced potential of the hESC present in largely differentiated populations to form teratomas could be due to signals arising from the differentiated cells leading to hESC death or differentiation and may be an additional important consideration in the assessment of the safety of these populations. In the two cases above the decrease in sensitivity appears to be of a similar magnitude However, it is possible that the sensitivity of the assay may vary depending on the specific differentiated cell population that is being analyzed and hence should be specifically determined for various differentiated cell populations.

In conclusion, we have presented detailed characterization of a quantitative, sensitive, easy-to-perform and monitor teratoma assay. Our results may serve as a basis for a standardized and uniform teratoma assay for the analysis of pluripotency. The assay may be also used for preclinical bio-safety analysis of pluripotent stem cell-derived differentiated therapeutic populations during the course of their development.

## Materials and Methods

### Cell Culture

hESC lines HES-1 and HES-2 [2] were cultured on human foreskin feeders in serum free medium as described [29]. The cells were passaged routinely as small clusters by treatment with 1 mg/ml collagenase type IV (Invitrogen, Carlsbad, CA). Foreskin fibroblasts [29] were cultured in 90% DMEM (Invitrogen) supplemented with 10% Fetal Calf Serum (FCS, Biological industries), 0.2 mM L-glutamine, 50 U/ml penicillin, and 50 µg/ml streptomycin. The cells were inactivated by treatment with 10 microgram/ml mitomycin C (MMC), dissociated into a single cell suspension with 0.05% trypsin-EDTA (Invitrogen), and frozen. HES-1 derived retinal pigment epithelium (RPE) cells were cultured as previously described [18].

### Characterization of Transplanted hESCs

Prior to transplantation a cell-sample was collected for karyotype analysis. In addition, FACS analyses were performed for detection of cell surface markers that are expressed in undifferentiated hESCs, using the following antibodies: Tra-1-60 (mouse monoclonal, clone MAB4360, Chemicon, Temecula, CA, 1∶100), Tra-1-81 (mouse monoclonal, clone MAB4381, Chemicon, 1∶100), and SSEA-4 (mouse monoclonal, clone MAB4304, Chemicon, 1∶100).

### Preparation of hESCs for Transplantation

On the day of transplantation hESCs were harvested and dissociated into a single cell suspension using 0.05% EDTA (Biological Industries, Beit-Haemek, Israel) .To ensure a single cell suspension the cells were filtered through a sterile filter. The filtered cells were centrifuged and the pellet was re-suspended in PBS (Invitrogen). To determine the number of viable cells, an aliquot of the hESCs was counted. Frozen MMC-treated foreskin fibroblasts were thawed and centrifuged. The pellet was re-suspended in PBS (Invitrogen), and viable cells were counted. For transplantation, various numbers of hESCs were combined with MMC-treated foreskin cells to a total of 1×10^6^. The cell-mixture was centrifuged and the pellet was re-suspended in PBS to a total volume of 50 µl. For negative controls, 1×10^6^ MMC-treated foreskin cells were transplanted. When the ROCK inhibitor Y-27632 (Calbiochem, Darmstadt, Germany) was included in the experiment, it was added to the cell mixture to a final concentration of 10 µM. The cells were kept for a minimal period at room temperature and transferred to the animal facility. The cell-mixture was combined with 50 µl undiluted cold Matrigel Basement membrane matrix (BD #354234, NJ, USA) immediately before transplantation. Pre-cooled pipettes, tips and syringes were used.

### Transplantation and Animal Observation

Five week old immune-deficient NOD/SCID mice were used for transplantation. They were maintained under non-specific pathogen-free (SPF) conditions, at the Hadassah Animal Facility. The cell-Matrigel mixture (100 µl) was injected into the subcutis of the right hind leg of the mice. The transplanted animals were observed routinely once a week, and tumor growth was measured with a caliper. They were sacrificed after development of tumors larger than 1 cm^3^ or following an observation period of 30 weeks. The sacrificed animals were subjected to general inspection. When tumors were observed at the injection site, they were collected. In addition, the liver, lungs and spleen tissues of the all animals were inspected for tumor invasion into other organs.

### Histological Analysis

The tumor containing tissues were fixed in 4% PFA, embedded in paraffin and serially sectioned into 6 micron sections. Every other section was mounted on slides (2–3 sections per slide, and 20 slides for each tumor). 4 slides, containing 8–12 sections from various parts of the tumor (slides #3, 8, 13 and 18) were stained with hematoxylin and eosin, and subjected to histological analysis by a certified pathologist. Large tumors were cut into halves before fixation, and the two halves were placed in the paraffin block so that the central parts of tumor (with the larger surface area) were faced up. To enable analysis of the different parts of large tumors, the sectioning was performed from the upper part of the paraffin block, containing the central parts of both halves of the tumor, towards the tumor’s edges.

### Statistical Analysis

Data is presented as mean ± SEM (standard error of mean). Kaplan-Meier analysis was performed using the GraphPad PRISM version 4.0 software.

### Ethics Statement

This study was performed in accordance with the recommendations of the Authority for Biological and Biomedical Models of the Hebrew University, Jerusalem. The protocol was approved by the Committee on the Ethics of Animal Experiments (Permit Number: MD-09-11663-4).

## Supporting Information

Figure S1 **Expression of pluripotency-associated cell surface markers in transplanted HES-1 cells. (A):** A representative FACS analysis of the expression levels of pluripotency-associated cell-surface markers, Tra-1-60, Tra-1-81, and SSEA-4 in transplanted HES-1 cells. **(B):** Average expression levels of Tra-1-60, Tra-1-81, and SSEA-4 in transplanted HES-1 cells from twelve transplantation experiments. Data presented as mean ± SEM.
(TIFF)

Figure S2 **Effect of the ROCK-inhibitor Y-27632 on Teratoma Formation.** Kinetics of teratoma formation after transplantation of decreasing numbers of undifferentiated HES-1 cells in the presence (dark gray) or absence (light gray) of the ROCK-inhibitor Y-27632. **(A):** Efficiency of teratoma formation **(B):** The average volume of the teratomas at the time of animals sacrifice. **(C):** The average time interval between transplantation and the detection of tumors. **(D):** The average time interval between transplantation and the experiment endpoint. Data presented as mean ± SEM.
(TIFF)

Table S1 **Teratoma Formation Kinetics after Transplantation of Various Defined Numbers of Undifferentiated HES-2 Cells.** Various numbers of undifferentiated HES-2 cells were mixed with MMC-treated foreskin fibroblasts (to a total of 1×10^6^ cells) and Matrigel, and transplanted s.c. into NOD/SCID mice. The transplanted animals were weekly monitored for the appearance of tumors, and for the progression of tumor size. The endpoint of the experiments was when the tumors reached a size of ≥ 1 cm^3^ or 30 weeks after transplantation. Data presented as mean ± SEM.
(DOC)
