# Supplementary figures and images for: Standardization of the Teratoma Assay for Analysis of Pluripotency of Human ES Cells and Biosafety of Their Differentiated Progeny
Source: PLoS One. 2012 Sep 25;7(9):e45532. doi: 10.1371/journal.pone.0045532 (PMC3458078; doi:10.1371/journal.pone.0045532)

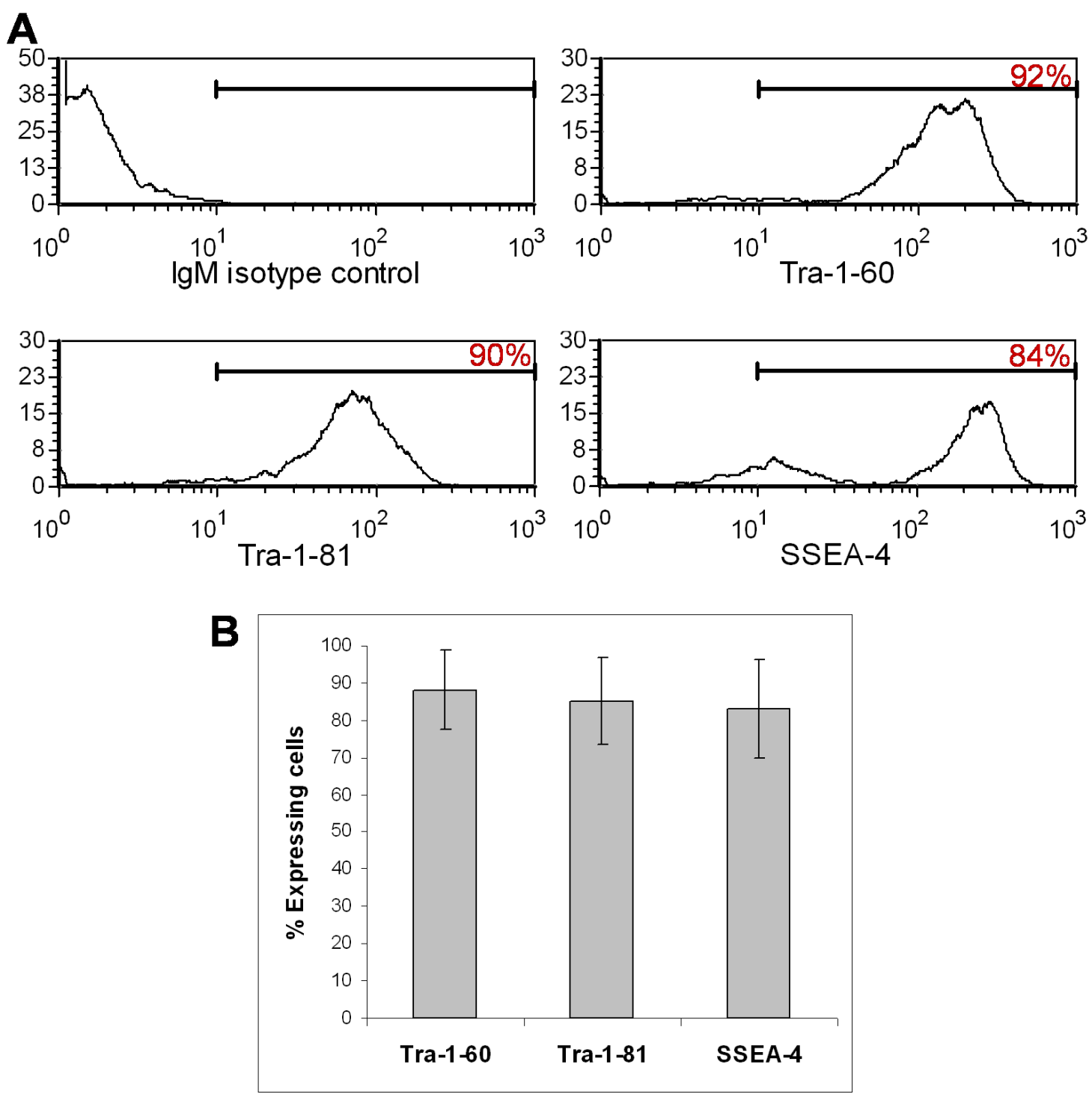

Supplement: Figure S1 — Expression of pluripotency-associated cell surface markers in transplanted HES-1 cells. (A): A representative FACS analysis of the expression levels of pluripotency-associated cell-surface markers, Tra-1-60, Tra-1-81, and SSEA-4 in transplanted HES-1 cells. (B): Average expression levels of Tra-1-60, Tra-1-81, and SSEA-4 in transplanted HES-1 cells from twelve transplantation experiments. Data presented as mean ± SEM. (TIFF) [file pone.0045532.s001.tiff]

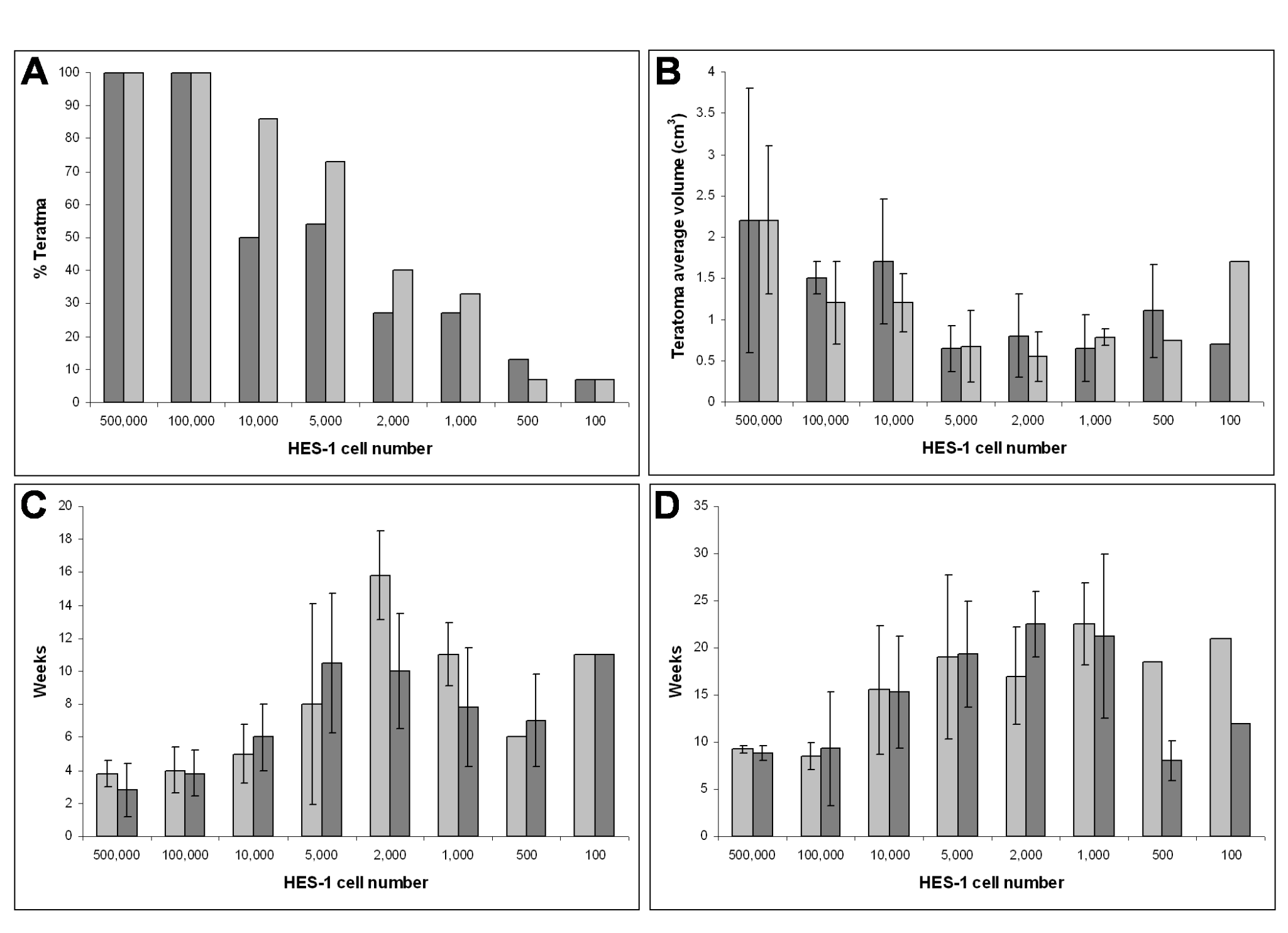

Supplement: Figure S2 — Effect of the ROCK-inhibitor Y-27632 on Teratoma Formation. Kinetics of teratoma formation after transplantation of decreasing numbers of undifferentiated HES-1 cells in the presence (dark gray) or absence (light gray) of the ROCK-inhibitor Y-27632. (A): Efficiency of teratoma formation (B): The average volume of the teratomas at the time of animals sacrifice. (C): The average time interval between transplantation and the detection of tumors. (D): The average time interval between transplantation and the experiment endpoint. Data presented as mean ± SEM. (TIFF) [file pone.0045532.s002.tiff]
