# Supplementary material for: Standardization of the Teratoma Assay for Analysis of Pluripotency of Human ES Cells and Biosafety of Their Differentiated Progeny
Source: PLoS One. 2012 Sep 25;7(9):e45532. doi: 10.1371/journal.pone.0045532 (PMC3458078; doi:10.1371/journal.pone.0045532)
Supplement: Table S1 — Teratoma Formation Kinetics after Transplantation of Various Defined Numbers of Undifferentiated HES-2 Cells. Various numbers of undifferentiated HES-2 cells were mixed with MMC-treated foreskin fibroblasts (to a total of 1×10^6^ cells) and Matrigel, and transplanted s.c. into NOD/SCID mice. The transplanted animals were weekly monitored for the appearance of tumors, and for the progression of tumor size. The endpoint of the experiments was when the tumors reached a size of ≥ 1 cm3 or 30 weeks after transplantation. Data presented as mean ± SEM. (DOC) [file pone.0045532.s003.doc]

| **Teratoma average volume (cm^3^)** | **Experiment average endpoint (weeks)** | **Teratoma average appearance (weeks)** | **Teratoma percentage** | **Teratoma number** | **Animal number** | **hES cell number** |
| --- | --- | --- | --- | --- | --- | --- |
| 0.9 ± 0.2 | 10.3 ± 4.5 | 2.3 ± 0.6 | 100% | 3 | 3 | **1x10^5^** |
| 0.9 ± 0.2 | 8.0 ± 1.4 | 4.8 ± 1.6 | 83% | 5 | 6 | **1x10^4^** |
| 0.8 ± 0.2 | 10.1 ± 3.6 | 4.3 ± 1.6 | 100% | 7 | 7 | **5x10^3^** |
| 0.9 ± 0.2 | 15.3 ± 4.2 | 7.0 ± 3.0 | 71% | 5 | 7 | **1x10^3^** |
| 0.7 | 22.0 | 10.0 | 17% | 1 | 6 | **1x10^2^** |
